# Effectiveness of Intermittent Fasting and Time-Restricted Feeding Compared to Continuous Energy Restriction for Weight Loss

**DOI:** 10.3390/nu11102442

**Published:** 2019-10-14

**Authors:** Corey A. Rynders, Elizabeth A. Thomas, Adnin Zaman, Zhaoxing Pan, Victoria A. Catenacci, Edward L. Melanson

**Affiliations:** 1 Department of Medicine, Division of Geriatric Medicine, University of Colorado Anschutz Medical Campus, Aurora, CO 80045, USA; ed.melanson@cuanschutz.edu; 2 Eastern Colorado Veterans Affairs Geriatric Research, Education, and Clinical Center, Denver, CO 80045, USA; 3 Department of Medicine, Division of Endocrinology, Metabolism and Diabetes, Department of Medicine, University of Colorado Anschutz Medical Campus, Aurora, CO 80045, USA; elizabeth.thomas@cuanschutz.edu (E.A.T.); adnin.zaman@cuanschutz.edu (A.Z.); vicki.catenacci@cuanschutz.edu (V.A.C.); 4 Department of Medicine, Anschutz Health and Wellness Center, University of Colorado Anschutz Medical Campus, Aurora, CO 80045, USA; 5 Department of Biostatistics and Informatics, University of Colorado Anschutz Medical Campus, Aurora, CO 80045, USA; zhaoxing.pan@cuanschutz.edu

**Keywords:** weight loss, alternate day fasting, meal timing, obesity

## Abstract

The current obesity epidemic is staggering in terms of its magnitude and public health impact. Current guidelines recommend continuous energy restriction (CER) along with a comprehensive lifestyle intervention as the cornerstone of obesity treatment, yet this approach produces modest weight loss on average. Recently, there has been increased interest in identifying alternative dietary weight loss strategies that involve restricting energy intake to certain periods of the day or prolonging the fasting interval between meals (i.e., intermittent energy restriction, IER). These strategies include intermittent fasting (IMF; >60% energy restriction on 2–3 days per week, or on alternate days) and time-restricted feeding (TRF; limiting the daily period of food intake to 8–10 h or less on most days of the week). Here, we summarize the current evidence for IER regimens as treatments for overweight and obesity. Specifically, we review randomized trials of ≥8 weeks in duration performed in adults with overweight or obesity (BMI ≥ 25 kg/m^2^) in which an IER paradigm (IMF or TRF) was compared to CER, with the primary outcome being weight loss. Overall, the available evidence suggests that IER paradigms produce equivalent weight loss when compared to CER, with 9 out of 11 studies reviewed showing no differences between groups in weight or body fat loss.

## 1. Introduction

The current obesity epidemic in developed countries is staggering in terms of its magnitude and public health impact. Healthy weight individuals (body mass index (BMI) of 18.5–25 kg/m^2^) are now the minority in the United States [1]. Medical spending attributable to overweight and obesity has been estimated to be over 90 billion dollars in the Unites States alone [2]. Reducing the daily calorie intake is the most widely prescribed strategy to induce weight loss [3]. Current guidelines recommend continuous energy restriction (CER; a daily energy deficit of ~500 or 750 kcals, or a 30% restriction from baseline energy requirements), along with a comprehensive lifestyle intervention, as the cornerstone of obesity treatment [4]. On average, this approach produces modest weight loss (5–10% sustained for ≥1 year) [4]. The magnitude of weight loss with CER is minimally influenced by variations in diet macronutrient content, especially during long-term follow-up [5,6,7,8,9,10]. Regardless of diet and macronutrient content, adherence to CER typically declines within 1–4 months [11]. As a result, the majority of individuals who lose weight with CER regain significant weight within 1 year [12,13,14]. 

Because of the relative ineffectiveness of traditional CER approaches for achieving and sustaining weight loss, there has been increased interest in identifying alternative dietary weight loss strategies. One such approach is prolonging the fasting interval between meals (i.e., intermittent energy restriction, IER). The premise of this approach is that individuals do not fully compensate during fed periods for the energy deficit produced during extended periods of fasting between eating bouts. Furthermore, these regimens may be easier to adhere to and maintain over time than CER. Finally, IER may lead to metabolic adaptations that favor a greater loss of fat mass, the preservation of lean mass, and a greater ability to sustain weight loss [15]. 

Various IER regimens have gained popularity in recent years as strategies for achieving weight loss and other metabolic health benefits [16,17,18]. These paradigms involve recurring periods with little or no energy intake (e.g., 16–48 h), with intervening periods of ad libitum food intake. Studies in rodents have demonstrated that IER strategies such as intermittent fasting (IMF; ≥60% energy restriction on 2–3 days per week, or on alternate days) and time-restricted feeding (TRF; limiting the daily period of food intake to 8–10 h or less on most days of the week) exert beneficial effects on the body composition, energy expenditure, and substrate oxidation. However, clinical studies comparing weight loss with IER regimens to traditional CER in adults with overweight and obesity are limited. 

The objective of this review is to summarize the current evidence for IER regimens as treatments for overweight and obesity. We first review the evidence from pre-clinical studies to provide a background on the purported mechanisms by which IER induces long-term benefits for body weight and composition. We then present a narrative review of randomized trials of ≥8 weeks in duration performed in adults with overweight or obesity (BMI ≥ 25 kg/m^2^) in which an IER paradigm (IMF or TRF) was compared to CER and the primary outcome was weight loss. We identified studies to include in this literature review by using specific search terms in PubMed and cross-referencing citations. The final goal of this review is to identify gaps in the current evidence base and outstanding scientific questions regarding IER strategies for weight loss.

## 2. Intermittent Energy Restriction (IER) Strategies Defined

**Figure 1:**
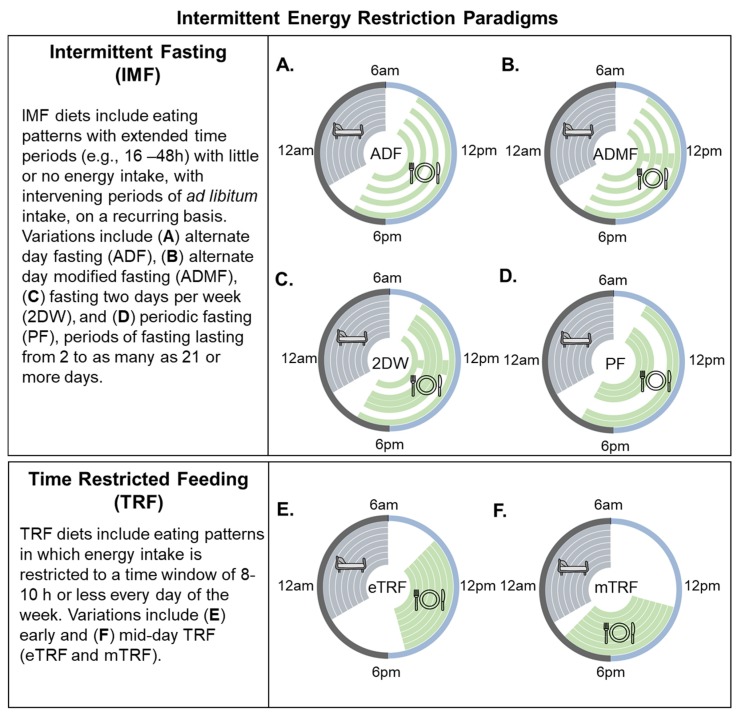
Popular variations of intermittent energy restriction. Within the circles, each ring represents a distinct 24 h day. Green shaded areas represent eating periods. Grey shaded areas indicate the sleeping time, and white circles/spaces indicate fasting periods. Intermittent fasting (IMF) is characterized by recurring periods (e.g., 16–48 h) with little or no energy intake. Many variations have been used to study the effects of IMF on body weight, including (**A**) alternate day fasting (zero calorie intake on fast days), (**B**) alternate day modified fasting (>60% energy restriction on fast days), and (**C**) fasting or modified fasting on two days per week (2DW). (**D**) Periodic fasting involves fasting for 2 to as many as 21 or more days. This IMF paradigm is acknowledged in the present review but will not be discussed as there are few studies in the literature. Time-restricted feeding (TRF) is characterized by eating patterns that are restricted to a short (<8–10 h) interval each day, such as during the (**E**) early or (**F**) middle portion of the day.

A major problem in the field is a lack of standardization of terminology to describe different intermittent energy restriction paradigms. Mattson et al. [15,19] have used intermittent fasting as the umbrella term to define “eating patterns in which individuals go extended time periods (e.g., 16–48 h) with little or no energy intake, with intervening periods of normal food intake, on a recurring basis”. They further “distinguish studies of short-term frequent fasting periods from studies of less frequent but longer fasting periods” by using the term periodic fasting (PF) to refer to IMF regimens with periods of fasting lasting from 2 to as many as 21 or more days. The term time-restricted feeding (TRF) is used as a subcategory of IMF to describe “an eating pattern in which food intake is restricted to a time window of 8 h or less every day”. The categorization of TRF as a type of IMF diet is problematic because TRF is fundamentally different from a complete day of fasting or even a modified fast day (generally defined as a day on which individuals consume up to 25% of daily energy needs). As will be discussed below, TRF is simply an appropriate eating pattern (eat during the day and not at night) that humans have veered away form in the past several decades. Rodent studies have shown that reducing the daily eating duration has beneficial effects on weight, body composition, and metabolism. Importantly, these effects are seen even without a reduction in daily energy intake [20,21,22]. For these reasons, we propose the use of the umbrella term IER, which has two distinct categories—IMF and TRF (see Figure 1).

Intermittent fasting regimens involve 60–100% energy restriction on fast days with ad libitum energy intake on fed days. Various IMF regimens have been proposed, with the most popular being alternate day fasting (ADF) and a regimen of fasting for two days per week (2DW). A common and appealing feature of IMF is that dieters do not have to restrict calories every day [17]. Weight loss likely occurs because individuals do not fully compensate on non-fasting days for the calorie deficit that occurs on fasting days [23,24,25,26,27]. Furthermore, the periodic nature of fasting may mitigate the constant hunger associated with CER [28]. For example, several studies using a modified fasting paradigm (~25% of energy requirements on fast days) have shown that hunger decreases [23,29] or remains unchanged [30,31] from the baseline over an 8–12 week IMF intervention. 

TRF refers to an eating pattern in which the food intake is restricted to a time window of 8–10 h or less every day. Recent studies in humans suggest that the median duration of eating is approximately 14.5 h per day, and the assumption that most individuals regularly undergo an “overnight fast” may thus be incorrect [32]. Eating “around the clock” has been suggested to have detrimental effects on health and body weight, leading to a desire to study time-restricted feeding as a weight loss strategy [15]. Limiting the eating duration may be an effective strategy to reduce the overall caloric intake; however, TRF does not necessarily have to involve caloric restriction. TRF is distinct from IMF because it involves an element of timing optimally aligned to the biological day.

## 3. Effects of IMF on Body Weight, Body Composition, and Metabolic Outcomes: Evidence from Preclinical Studies

A key research question is whether IMF elicits benefits for body weight and peripheral/tissue-specific metabolism that are at least comparable to CER. This question is challenging to rigorously address in human clinical trials because of issues with adherence to energy intake prescriptions and the lack of a criterion method for measuring the actual free-living intake. In contrast, manipulating the energy intake and determining the actual intake can be accomplished with a high degree of accuracy in animal models. Alternate day fasting has been the most thoroughly studied IMF protocol in laboratory rodents [15,33,34,35]. A few studies have used a CER protocol for 3–6 consecutive days, followed by a ‘break’ period of ad libitum feeding (e.g., 4 days of a CER followed by 3 days of ad libitum feeding) [15,19]. However, for the purposes of this review, we will only consider preclinical studies of ADF as this protocol is most consistent with the definition of IMF that we have provided here and with previous definitions proposed by Mattson et al. [15,19].

Compared to ad libitum fed animals, ADF appears to improve several circulating and tissue-specific biomarkers associated with the metabolic health status (for reviews, see refs [15,19]). In mice, ADF mitigates the adverse effects of ad libitum high-fat feeding, resulting in a lower fat mass, and thus reducing plasma glucose, insulin, and leptin levels, and improving glucose tolerance [15,19]. To our knowledge, only two preclinical studies (Anson et al. [34] and Mager et al. [33]) have performed head-to-head comparisons of ADF and CER regimens and measured longitudinal weight change and metabolic outcomes. Anson et al. [34] studied mice over a period of ~20 weeks assigned to one of four groups: mice fed ad libitum; mice provided ad libitum access to food every other day (i.e., ADF); mice provided with a limited daily food allotment of 60% of that eaten by the ad libitum fed animals (i.e., CER); and pair-fed mice that were provided a daily food allotment equal to the average daily intake of mice in the ADF group. The fourth group provided an opportunity to isolate the effects of total calorie intake versus ADF. Over ~20 weeks, the ADF mice compensated for periods of fasting by almost doubling the food intake on fed days, thus gaining weight at rates similar to the ad libitum-fed mice. As expected, the pair-fed mice gained an identical amount of weight to the ADF and ad libitum-fed mice, while the CER mice maintained a significantly lower weight. Interestingly, despite different body weights and levels of intake, fasting glucose and insulin concentrations were improved to a similar extent in the ADF and CER groups, an effect that was not observed in the pair-fed or ad libitum-fed mice. This is in contrast to a study conducted by Mager et al. [33] that reported unchanged glucose concentrations in Sprague-Dawley rats following a period of ADF, and reduced glucose concentrations in a group of rats following a 40% CER regimen. Similar to the results of Anson et al. [34], the body weight remained lower in the CER rats compared to the rats that received ADF. 

An additional study worth noting (Gotthardt et al. [35]) compared ADF combined with either a low- or high-fat diet to groups of mice that were fed low- and high-fat diets ad libitum. This study is relevant to human weight loss because animals were placed into the diet groups after a period of weight gain. C57/BL6 mice were fed a high-fat diet (HFD; 45% fat) ad libitum for 8 weeks to promote an obese phenotype and mice were then divided into four groups that were either maintained for four weeks on an ad libitum high-fat diet (HFD), maintained on an ad libitum low-fat diet, received a high-fat diet every other day (ADF-HFD), or received a 10% low-fat diet every other day (ADF-LFD). At 4 weeks, body weights were significantly lower in the ADF-HFD group (~13% reduction) and ADF-LFD group (~18% reduction) compared with the HFD group. All three diet groups had a statistically similar energy intake during the intervention. Mice on the ADF-LFD maintained a ~12% higher lean mass over 4 weeks compared to the ad libitum LFD and HFD groups. Only the ADF-LFD group had improved glucose tolerance at 4 weeks compared to the other groups. Although a limitation of the study is that it lacked a diet-specific calorie-restricted group, the results suggest that the dietary macronutrient composition potentially modifies the metabolic response to ADF. 

Taken together, the effects of ADF on body weight and glucose (at least in rodents) seem to depend on a number of factors, such as the energy and macronutrient content of the diet. Importantly, in contrast to humans, rodents fed normal chow every other day tend to fully compensate on fed days for the negative energy balance incurred on fast days [34] (see next section). The lack of well-controlled preclinical studies comparing ADF to CER represents a key evidence gap that could be addressed in future studies by including both CER and ADF groups and ideally, animals pair-fed to the IMF group, as was done in the work conducted by Anson et al. [34]. Future studies should also carefully consider how the CER group receives its daily allotment of food. Calorically-restricted rodents tend to eat all of their food as soon as it is made available (e.g., 3–6 h time window) [36]. This is a confounding factor that adds a timing component to the reduction in calories and extends the fasting duration, potentially rendering the CER group indistinguishable from the ADF animals.

## 4. Effects of TRF on Body Weight, Body Composition, and Metabolic Outcomes: Evidence from Preclinical Studies

In contrast to IMF, there have been no preclinical studies directly comparing TRF regimens to CER in the context of weight loss, so it is unknown whether this form of IER differentially impacts metabolic responses to weight loss. Therefore, we will briefly address the studies that have examined metabolic responses to TRF compared to an energy-matched ad libitum feeding group (i.e., no time restriction). 

Panda et al. [20,21,22] have investigated a diet paradigm where rodents fed an HFD ad libitum are compared to rodents allowed to consume the same number of calories within a restricted time interval of 8–10 h aligned to the active period. In these studies, TRF mice are protected from diet-induced obesity and have increased energy expenditure and fat oxidation. One hypothesized mechanism to explain the attenuated weight gain despite the high-fat feeding in TRF mice appears to be optimizing the alignment of the feeding interval to the appropriate circadian time. Molecular circadian “clocks” act together to promote energy intake mostly during an organism’s active phase [37]. This circadian control of energy intake involves a primary clock in the suprachiasmatic nuclei of the hypothalamus that synchronizes to the solar light–dark cycle and secondary clocks located in other hypothalamic and brainstem regions. Rhythms in central metabolic hormones, circulating nutrients, and neural inputs transmit timing cues to peripheral organs which allow peripheral organs to anticipate feeding and fasting periods and prime metabolic responses appropriately. 

Another mechanism through which TRF may improve body weight regulation is that of an extended fasting duration. Similar to what occurs in IMF, extending the daily fasting duration likely promotes the mobilization of free fatty acids (FFA), increases fat oxidation, and increases the production of ketones [22]. Whether these effects are potentiated when TRF is combined with CER and whether this combination is superior to CER alone are open research questions in both pre-clinical and clinical research.

Finally, one study has suggested that TRF appears to affect host metabolism by altering the gut microbiome to one that is less obesogenic [38]. TRF appears to restore the diurnal variation in several families of bacteria that are involved in nutrient absorption when animals are fed a high-fat diet [39]. In the study by Panda et al. [38], TRF restored diurnal variation in the Lactobacillus and Ruminococcacea families, which have been hypothesized to protect against the metabolic consequences of obesity. TRF mice also excreted more breakdown products of complex carbohydrates (e.g., xylose and galactose), which can only be degraded by gut microflora [39]. These data were interpreted to suggest that complex carbohydrates are less easily absorbed in TRF mice compared to high-fat fed mice. 

## 5. Current Evidence for IMF as a Weight Loss Strategy: Evidence from Humans? Clinical Studies

We reviewed all randomized weight loss studies performed in adults with overweight or obesity (BMI ≥ 25 kg/m^2^) that involved a comparison of IMF to CER. Studies included in the review were ≥8 weeks in duration, and the IMF paradigm prescribed ≥60% energy restriction on “fast” days, with intervening “fed” days of ad libitum intake or intake at or above daily energy needs (see Table 1). We did not include studies that utilized ≤60% energy restriction on fast days to remain consistent with prior definitions of IMF in the literature [15]. Interventions of less than 8 weeks were not included because 8 weeks is not a sufficient duration to provide meaningful, clinically relevant information in regard to weight loss. Eleven studies met our criteria and are summarized in Table 1. Because there are no commonly accepted definitions and terminology used to describe the various IMF paradigms, a description of each intervention is provided in the table. Harvie et al., 2011 [40]; Harvie et al., 2013 [41]; Carter et al., 2016 [42]; Carter et al., 2018 [43]; Schübel et al. [44]; Conley et al. [45]; and Sundfør et al. [46] all evaluated various forms of the 2DW IMF diet (either 25% [40,44] or 30% [41] of energy intake (EI) or specific calorie goals on 2 restricted days per week [42,43,46], with no restriction on the other 5 days). Catenacci et al. [25] evaluated zero-calorie alternate day fasting (100% energy restriction on fast days alternating with ad libitum intake on fed days), while Varady et al. [47], Trepanowski et al. [48], and Hutchison et al. [49] evaluated alternate day modified fasting (ADMF) (25% [47,48] and 37% [49] of energy requirements on fast days alternating with either ad libitum intake on fed days [47] or the provision of 100–145% [48,49] of EI on fed days). These studies generally enrolled participants who were physically inactive or engaged in light or low amounts of physical activity at the baseline. All studies had a majority of female participants, with the exception of Schübel et al. [44] and Sundfør et al. [46], both of which included 50% males, and Conley et al. [45], which included only older male war veterans. Four studies—Varady et al. [47], Catenacci et al. [25], Trepanowski et al. [48], and Hutchison et al. [49]—provided meals to study participants during some or all of the intervention; all other studies gave energy intake goals but allowed participants to eat their own food. 

The level of dietary counseling and contact with dietitians was variable across studies. Studies that provided food generally did not include dietary counseling [25,47], while other studies provided weekly [49] or biweekly [40,41,42,43,44,46] dietary counseling in person or over the phone. A few studies did not provide exercise recommendations during the intervention [42,43,49], but most studies recommended that participants maintain their habitual physical activity levels [25,40,44,46,47,48]. Only the Harvie et al., 2013 [41] study provided the recommendation of gradually increasing the frequency and intensity of exercise. The only study that objectively measured physical activity (Trepanowski et al. [50]) found no change in the number of steps per day in any group.

Overall, the available evidence suggests that IMF paradigms produce equivalent weight loss when compared to CER, with 9 out of 11 studies reviewed showing no significant differences in weight or body fat loss between IMF and CER groups. Our findings are consistent with a recent systematic review and meta-analysis conducted by Harris et al. [51] that included four studies comparing IMF to CER published through November 2015. We excluded two studies included in that systematic review and meta-analysis (Hill et al. [52] and Viegener et al. [53]) due to the fact that the regimens did not include intervening days of ad libitum food intake, but alternated periods of significant (600–900 kcal/day) with moderate (1200–1500 kcal/day) energy restriction. In the Harris et al. review, the authors found no significant difference between IMF and CER (−1.03 kg, 95% CI −2.46 kg to 0.40 kg). In the present review, the only studies that showed differences in weight or body fat loss were Harvie et al., 2013 [41] and Hutchison et al. [49]. The Harvie et al., 2013 study included a 2DW IMF regimen which required two consecutive days/week of either a low-carbohydrate diet (70% energy restriction and 40 g carbohydrate) or a less restrictive low-carbohydrate 2DW IMF diet that allowed ad libitum protein and monounsaturated fatty acids. The 2DW IMF diets were compared to an isocaloric 25% CER Mediterranean-type diet. While there was no significant difference in weight loss between the groups, there was a greater loss of body fat (measured using bioimpedance) with both 2DW regimens compared to CER over 3 months: mean change in body fat was −3.7 kg (95% CI −2.5, −4.9) for the low-carbohydrate 2DW group, −3.7 kg (95% CI −2.8, −4.7) for the low-carbohydrate 2DW plus ad lib protein group, and −2.0 kg (95% CI −1.0, 3.0) for the CER group. The Hutchison et al. [49] study included an ADMF group that was provided a diet at 70% of the calculated baseline energy requirements per week (ADMF 70), an AMDF diet at 100% of the calculated baseline energy requirements per week (ADMF 100), and a CER group at 70% of the calculated baseline energy requirements daily (CER). The two ADMF groups were provided meals on their fed days (~100% energy requirements for ADMF 70 and ~145% energy requirements for ADMF 100) and were asked to consume breakfast (32% of energy requirements for ADMF 70 and 37% in ADMF 100) before 8 am on 3 nonconsecutive fast days per week, followed by a 24 h fast until 8 am the following day. ADMF 70 produced greater weight loss (−5.4 ± 0.5 kg) and fat loss (−3.9 ± 0.4 kg) compared to both CER (−3.9 ± 0.4 kg; −2.8 ± 0.4 kg) and ADMF 100 (−2.7 ± 0.5 kg; −2.3 ± 0.4 kg). Importantly, both the ADMF 100 and ADMF 70 groups ate less than provided on fed days, resulting in an overall average weekly deficit of ~9% and ~2% more than prescribed, respectively, such that energy restriction in ADMF 70 was greater than CER. This spontaneous energy restriction on fed days was also observed in Harvie et al., 2013 [41] and Trepanowski et al. [48], and has been hypothesized to be a benefit of IMF [54]. Schubel et al. [44] showed a trend toward greater weight loss with 2DW IMF compared to CER (log relative weight change −7.1 ± 0.7% vs. −5.2 ± 0.6%, *p* = 0.053). Five studies (Harvie et al., 2013 [41], Catenacci et al. [25], Schubel et al. [44], Trepanowski et al. [48], and Sundfør et al. [46]) included maintenance and/or follow-up phases, with relatively minimal contact with participants for 4–26 weeks. In general, there was no difference in weight regain between IMF and CER groups over these follow-up periods. 

## 6. Current Evidence for TRF as a Weight Loss Strategy: Evidence from Humans? Clinical Studies

There is some evidence to suggest that the timing of meals may impact weight loss. In two weight-loss interventions, individuals who self-reported consuming more calories in the morning compared to the evening lost more weight, even though both the energy intake and self-reported physical activity were similar [55,56]. In addition, food intake at night (as seen with shift work) is linked to obesity, independent of energy intake [57,58,59]. However, only a few human trials of TRF (eating window ≤8–10 h for ≥8 weeks) have been conducted in individuals with overweight/obesity with weight loss as an outcome, and none of these trials compared TRF to CER. Gill and Panda [32] studied eight overweight men and women with a habitual eating duration >14 h who were asked to restrict EI to a self-selected 10–12 h window. Although participants were not instructed to reduce EI, the restricted eating window resulted in a ~20% reduction in the daily caloric intake estimated using photographic food records. Subjects lost weight (−3.3 kg, 95% CI −5.6 to −0.9 kg) and maintained the weight loss at 1 year. Interestingly, they also reported subjective improvements in sleep satisfaction, less hunger at bedtime, and increased energy levels. In a study by Gabel et al. [60], 23 obese subjects were asked to restrict feeding (without calorie counting) to an 8 h window (10:00 to 18:00) for 12 weeks, and weight loss was compared to historical controls. TRF resulted in modest weight loss (−2.6 ± 0.5%) compared to the control (no change). In a study by Antoni et al. [61], 16 men and women (BMI 20–39) were randomized to TRF without energy restriction or control for 10 weeks. The TRF group was asked to delay the first energy intake and advance the last energy intake of the day by 1.5 h each. Participants in the TRF group successfully reduced the eating windows by approximately 4.5 h and reduced the overall energy intake compared to the control group. The TRF group had a significant reduction in body fat percentage (1.9 ± 0.3%), as measured by bioimpedance, but there was no significant change in body weight in either group. 

## 7. Are the Metabolic Benefits of IMF and TRF in Clinical Studies Solely Due to Caloric Restriction and Weight Loss?

In general, the available evidence suggests that IMF offers little to no advantage over CER for improvements in risk factors for cardiometabolic disease (See Table 2). Small reductions in total cholesterol (TC), low-density lipoprotein (LDL) cholesterol, triglycerides, and fasting glucose and insulin with IMF have been reported in several studies [25,40,41,42,44,47,48,49,50,62], but these changes are generally similar in magnitude to those observed with CER. Because weight loss was similar in most studies, it appears that the magnitude of weight loss, rather than dietary treatment, is the most important factor driving these changes. Although Hutchison et al. [49] reported that decreases in LDL and total cholesterol were greater with ADMF (after adjusting for weight loss), the magnitude of these differences was small [49]. High-density lipoprotein (HDL) levels remained unchanged in most studies, and when significant changes were observed, the changes were not different from those observed with CER. Several studies reported improvements in insulin resistance measured using the Homeostatic Model Assessment of Insulin Resistance (HOMA) model [40,41,62] with 2DW IMF, and one study demonstrated greater improvements in insulin sensitivity measured using the euglycemic clamp method with ADMF [49], but ADMF participants lost slightly more weight than those of CER. When observed, the changes in HOMA IR appear to be driven by small decreases in fasting insulin. However, metabolic measurements were secondary outcomes in most of these studies, and thus, it is likely that these studies were underpowered to detect differences between dietary treatments. Nonetheless, the relatively small changes in these measures suggest that any observed changes or differences between groups have minimal clinical importance. It should be noted that most studies to date have enrolled relatively healthy participants with overweight and obesity. To our knowledge, only one small study has been performed comparing 2DW IMF to CER in individuals with type 2 diabetes [42]. In that study, HbA1c was significantly reduced after the intervention (−0.5% ± 0.2% in the CER group and −0.3% ± 0.1% in the 2DW group), but did not differ by intervention group. Therefore, whether IMF (2DW or another variation) would have greater benefits in individuals with more adverse metabolic profiles remains an area of future investigation.

An important issue to consider in study design is the timing of measurements relative to the fasting periods. Most studies have obtained samples for metabolic measurements on the day following a fed day. The one exception is the study of Hutchison et al. [49], in which samples were obtained following both a fed and fasted day. Ironically, clamp-measured insulin sensitivity tended to worsen in ADMF when measured the day after a fast. This effect could possibly be due to the acute elevation in fasting FFA following a fast day, which would be expected to impair insulin sensitivity [63]. Nonetheless, this study highlights some of the challenges of performing laboratory measures in studies comparing IMF to CER. To our knowledge, no study has used ambulatory measures (e.g., continuous glucose monitoring) when comparing IMF to CER. Performing such studies in individuals with impaired fasting glucose or type 2 diabetes is warranted.

It is also worth noting that a majority of the participants in these studies were pre-menopausal women, and there is no indication that attempts were made to account for the potentially confounding effects of phases of the menstrual cycle. Because some of these outcomes are known to vary across phases of the menstrual cycle, it is suggested that this potential confounding effect be controlled in studies where metabolic outcomes are being assessed.

Data regarding the metabolic effects of TRF in humans are limited. The study by Gabel et al. [60] showed a reduction in systolic blood pressure in the TRF group versus controls, but weight loss was greater in the TRF group. There were no differences in the diastolic blood pressure, body composition, cholesterol, homocysteine, fasting glucose, or insulin. Antoni et al. [61] showed a significant diet x group interaction for the change in fasting glucose, but this was driven by elevated glucose levels in the control group at the end of the intervention period. No changes in LDL cholesterol were observed.

## 8. Limitations of Previous Clinical Studies and Evidence Gaps

Despite the popularity of IMF and TRF as weight loss strategies, it is notable that only eleven randomized trials have compared IMF with CER, and no randomized trials have compared TRF to CER. In addition, many of the IMF trials provided participants in both IMF and CER arms with some [47,48,49] or all [25] of their food, which significantly limits the applicability to a real-world setting. Moreover, while current obesity treatment guidelines recommend a comprehensive lifestyle intervention consisting of on-site, high-intensity (≥14 sessions in 6 months) behavioral support provided in individual or group sessions by a trained interventionist [4], none of the studies we reviewed provided behavioral support for IMF meeting these criteria. The duration of the interventions ranged from only 8 to 26 weeks, so the efficacy of IMF over a longer duration, or during weight loss maintenance, has not been well-evaluated. Finally, none of these studies evaluated the effects of IMF or TRF on appetite (appetite-related hormones or hypothalamic expression of orexigenic peptides), physical activity, or exercise, nor have they assessed sex differences. Clearly, well-designed and longer-term randomized trials are needed to evaluate the effectiveness of IMF compared to the current dietary approach to weight loss (CER), when these approaches are delivered in a real-world setting with guideline-based behavioral support.

While it is well-recognized that adherence to weight loss interventions is the greatest predictor of weight loss success [11], data regarding adherence and acceptability of the IMF and TRF interventions in these studies are limited and variable. In addition, studies present adherence data in variable ways (i.e., percentage vs. absolute number of days adherent to energy restriction, vs. absolute percent energy restriction), which makes direct comparisons across studies difficult. Two studies provided no data regarding adherence to the prescribed diets [42,47]. Most of the IMF studies used seven-day diet records [40,41,44,46,48,49] or diet checklists [43] to assess adherence, with the exception of Catenacci et al. [25] and Hutchison et al. [49], which provided food and assessed adherence via food return. In a subset of participants, Trepanowski et al. [48] assessed dietary adherence using doubly-labeled water and reported no statistically significant difference in the percent energy restriction between the ADMF and CER by month 6 of the intervention. In other studies, self-reported adherence to the fast days was variable, with the percent of potential restricted days ranging from 43% in Harvie et al. [40] to 97% in Carter et al. [43] at 3 months. Studies with longer-term follow up data showed that self-reported adherence to the fast days dropped to between 21% [44] and 44% [43] at one year. Two studies provided no data regarding acceptability of the IMF intervention [47,49], but most studies assessed acceptability of the intervention via data on major adverse events and drop-out rates, which were generally found not to differ between groups [25,40,41,42,43,45]. However, Trepanowski et al. [50] reported a higher dropout rate in the ADMF group (38%) vs. CER (29%), with more participants in the ADMF group reporting dissatisfaction with the diet as a reason for withdrawal. Harvie et al. [40] reported that fewer participants in the 2DW IMF group planned to continue the diet at the end of the study compared to the CER group (58% vs. 85%). Sundfør et al. [46] found that participants in the 2DW IMF group reported more hunger, more adverse events, less favorable changes in diet nutritional composition and eating behavior [64], and greater measured weight regain than participants in the CER group.

In the TRF studies, self-reported adherence (number of days adherent to the eating window based on diet diaries) was 62.5% and 80% in studies by Antoni et al. [61] and Gabel et al. [60], respectively. However, in the study by Gill and Panda [32], it was reported that all participants reduced their eating duration (as measured with app-based photographic food records). However, no data were presented regarding the number or percentage of days that participants adhered to their eating windows. While Gill and Panda [32] reported that all participants expressed an interest in continuing the TRF regimen after the conclusion of the study, participants in the Antoni et al. [61] study rated the regimen with an average difficulty score of 7/10 (10 = extremely difficult to stick to the regimen every day), and 57% felt that they could not have maintained the TRF protocol beyond the 10 week intervention. Therefore, more rigorous, objective methods to assess the adherence and acceptability of IMF and TRF as long-term weight loss regimens are needed, as well as efficacy analyses that take adherence into account.

## 9. Future Directions and Outstanding Questions

### 9.1. Is IMF a Durable Weight Loss Strategy? 

Most, if not all, studies suggest that IMF paradigms are safe and tolerable and produce 3–8% weight loss in adults with overweight or obesity. However, our review highlights several limitations of the current evidence base. We only identified eleven randomized controlled trials greater than 8 weeks in duration that have compared IMF to CER with weight loss as a primary outcome. Nine of these studies report no significant differences in weight or body fat loss between IMF and CER, and in the two studies that found significant differences, they were relatively modest. Generally, these studies have not been sufficiently powered to show equivalence in weight loss or to detect differences in body composition. The majority of studies are less than 26 weeks in duration and no studies to date have included a true follow-up period, leaving open the question of whether IMF is a durable weight loss strategy over longer periods of time. Future studies could provide ongoing behavioral support and perform frequent measures of weight and clinical outcomes following the intervention to determine whether IMF improves weight loss maintenance. 

### 9.2. Can We Predict Who Will Be Successful on an IMF or TRF Diet Compared to CER?

It is well-documented that there are substantial inter-individual differences in response to a wide range of obesity treatments. These differences are likely due to a combination of behavioral, biological, environmental, and psychosocial factors. Despite this, we currently lack the data to comprehensively identify the factors or interactions of factors that influence the response to obesity treatments. The Accumulating Data to Optimally Predict Obesity Treatment (ADOPT) Core Measures Project sponsored by the National Institutes of Health, Heart, Lung, and Blood Institute (NIH-NHLBI) provides the framework to explore factors which may contribute to variability in the weight loss response in obesity interventional trials [65,66,67,68]. The ADOPT working group has selected a list of core measures within four major domains: behavioral, biological, environmental, and psychosocial. The collection of data on these ADOPT core measures spanning the four interdisciplinary domains may provide more comprehensive insight into how these factors impact obesity treatment responses. Future IMF and TRF studies should consider including as many of the ADOPT core measures as practical to examine predictors, mediators, and moderators of responses to these obesity treatments, which may provide insight to match the diet type to the individual to enhance success.

### 9.3. Is TRF Alone a Durable Weight Loss Strategy and does TRF Enhance Weight Loss When Combined with CER?

Restricting the energy intake to a short window during waking hours and extending the length of the overnight fast may be a practical and useful strategy for promoting weight loss and weight maintenance. However, only three small studies have evaluated whether TRF alone produces weight loss in overweight adults [32,60,61]. These studies have limitations because one lacked a control group [32], one utilized a historical CER control group [60], and one compared TRF to a no intervention control [61]. No randomized weight loss studies to date have compared TRF to CER. In addition, the potential benefits of adding TRF to a CER weight loss program have yet to be evaluated in a well-controlled clinical trial. Future studies are needed to evaluate the effectiveness of TRF alone and TRF within the context of a behavioral weight loss intervention that includes a moderately reduced calorie diet and behavioral support to facilitate adherence.

### 9.4. How Do IMF and TRF Impact Components of Energy Balance and Macronutrient Oxidation in Humans?

No existing human studies comparing IMF or TRF to CER have objectively quantified the impact of these diets on the total daily energy expenditure or physical activity energy expenditure. While Trepanowski et al [50] did use activity monitors to measures steps and doubly-labeled water to calculate the percentage energy restriction, they did not present data regarding physical activity or the total daily energy expenditure. Only one study of ADF compared to CER has included measures of the resting metabolic rate [25]. Very few studies have addressed the influence of IMF or TRF on objective measures of energy intake, appetite, and hormones related to appetite [30,48,69]. Future studies should include accelerometer-based measures of physical activity to determine how IMF and TRF impact sedentary behavior and other domains of activity (e.g., light and moderate to vigorous physical activity). Doubly-labeled water could be used to measure the total daily energy expenditure and whole-room calorimetry could inform on how IMF and TRF influence macronutrient oxidation. Finally, it will be important to measure components of energy balance on both fed and fast days in future studies of IMF.

### 9.5. How Do We Define Meal Timing and What Is the Optimal Eating Window in Studies of TRF?

Most studies of TRF have used an arbitrary clock time to characterize the timing of food intake (e.g., 8 a.m. to 2 p.m.). However, this approach does not consider meal timing in relation to the internal circadian timing system. This is important because the association between BMI and the timing of food intake strengthens considerably when considering the timing of food intake in relation to the internal circadian time (e.g., dim-light melatonin onset) [58,70]. Furthermore, the internal circadian time and sleep/wake cycle are more tightly correlated than circadian time and external clock time. Because obtaining measures of dim-light melatonin is not practical in large clinical trials, future studies could prescribe the time of food intake relative to the sleep/wake cycle as a proxy for circadian time. Future studies should also consider or at least measure the chronotype (e.g., morning vs. evening types), which is the preference of timing of various behaviors such as sleep. The chronotype has been observed to modify the relationship between meal timing and obesity [71]. Interestingly, a recent study found that a ‘chronotype-adjusted diet’ was more effective than a traditional hypocaloric diet in terms of improving anthropometric parameters [72].

There is currently no consensus regarding whether the TRF eating window should be aligned to the early or middle to late part of the day. Rodent studies of TRF have opted to align the 8–10 h feeding window to the middle of the active phase [22], while human studies have either aligned the feeding window to the early to mid-part of the day [60,69,73,74,75] or have allowed participants to self-select a window [32]. The timing of food intake may modify the metabolic response to a TRF intervention, as observational studies have shown that later eating behaviors are linked to obesity and there appears to be reduced weight loss effectiveness in dieters who consume the main meal later in the day [56]. On the other hand, an early TRF regimen might be more difficult to adopt because it is less aligned with the social schedule. For example, some people might find it challenging to not eat dinner with their family if on a strict early TRF diet.

### 9.6. How Do Fasting and the Timing of Meals Impact the Temporal Organization of Behaviors such as Sleep and Activity across the 24 h Cycle?

Altering the timing of meals or omitting/partially omitting the food intake on some days but not others is likely to influence the temporal patterns of other behaviors across the day, such as activity and sleep. However, a major limitation is a lack of standard methodologies for the simultaneous measurement of 24 h patterns of food timing, activity, light exposure, and sleep. Identifying appropriate tools to assess the timing of behaviors pre, post, and during an intervention will be necessary to evaluate the compliance and feasibility. In addition, obtaining information about the timing of sleep and activity is likely to provide important mechanistic insight into the health benefits of IMF and TRF beyond weight loss. Accelerometers, wrist actigraphy, and detailed sleep logs are obvious candidates for collecting data on activity, sleep, and light exposure. However, there is no standard method for collecting data on the timing of energy intake. Current choices include asking participants to record clock times using traditional dietary assessment tools such as 24 h dietary recalls, food timing questionnaires (e.g., The Meal Pattern Questionnaire [76] or Night Eating Syndrome Questionnaire [77]), picture-based smartphone applications, and perhaps continuous glucose monitoring [78]. Limitations to these approaches include recall bias, the lack of validation, participant burden, analytic burden, and potential issues with scalability for large-scale clinical trials. 

**Figure 2:**
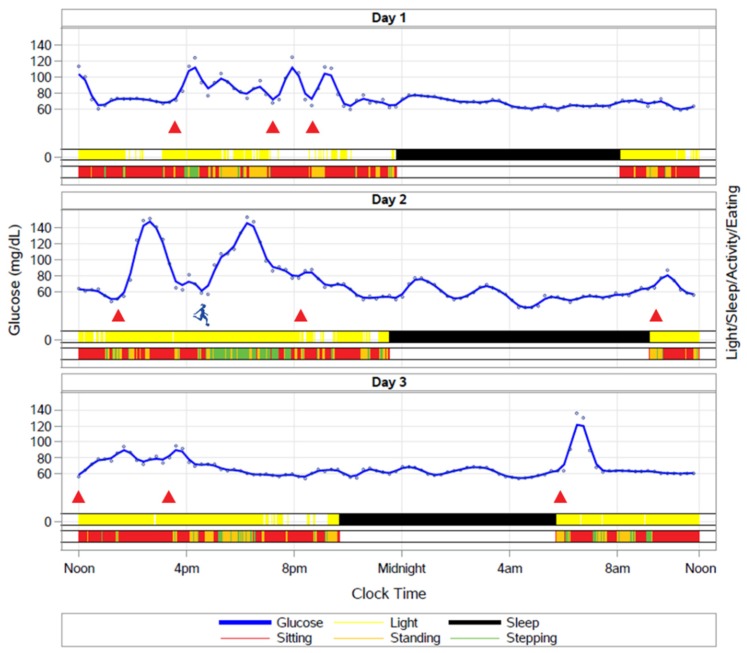
How IMF and TRF impact daily behavioral rhythms is entirely unknown, but will be important for understanding the durability of these interventions. Wearable devices such as activity monitors, light sensors, and continuous glucose monitors provide an opportunity to capture free-living behavior. Including these measures in clinical studies may help to identify phenotypes of individuals who are likely to benefit the most from fasting or timed feeding.

It might be possible to derive information about daily eating patterns by combining existing wearable technologies. In Figure 2, we provide an illustration of meal timing, activity, light exposure, and sleep patterns over 3 d using an activPAL accelerometer, Phillips Actiwatch Spectrum wrist watch, cell phone application, and continuous glucose monitor. The glucose excursions correspond to the food intake times captured by a picture-based cell phone application. By integrating information about sleep timing and activity (sitting, standing, and stepping), machine learning and other analytical techniques could be used to improve the ability to estimate meal timing from continuous glucose monitor (CGM) traces. For example, on Day 2, the participant has a meal around 2 pm that is associated with a glucose excursion. A similar glucose excursion occurs at 4 pm, but as a result of an intense soccer match rather than a meal. Improving the ability to detect meal patterns with CGM would drastically reduce the participant burden associated with questionnaire or cell phone-based approaches to determining food intake patterns. 

## 10. Conclusions

In this review, we have summarized the current evidence for various intermittent energy restriction regimens (IMF and TRF) as treatments for overweight and obesity. In addition, we have identified gaps in the current evidence base and outstanding scientific questions regarding intermittent energy restriction strategies for weight loss. Although IMF diets do not seem to produce greater weight loss than CER, there still exists a need to determine whether IMF influences body composition or metabolic parameters. Studies have not been sufficiently powered to detect differences in these outcomes. It will also be important to conduct larger clinical trials aimed at determining whether it is possible to predict who will be most successful with IMF versus CER. With regard to TRF, we are awaiting the first randomized clinical trials to determine how weight loss with TRF alone or TRF in combination with CER compares to CER alone. Finally, the nutrition field should work towards standardized methods for monitoring the timing of food intake in studies of IMF and especially TRF. It will also be important to characterize relationships between the timing of dietary intake and other aspects of behavior that are linked to health (e.g., sleep and physical activity). 

## Figures and Tables

**Table 1:** Randomized clinical weight loss studies with ≥8 week duration in individuals with overweight/obesity comparing an IMF paradigm to a calorie-restricted control group.

Reference	N	Age ^b^ (Years)	BMI (kg/m^2^)	Intervention Duration	Analysis	Interventions	Weight Loss ^a^	Attrition ^a^
Harvie et al., 2011 [40]	107 100% Female	30–45	24–40	26 weeks	Intent-to-Treat	2DW 25% overall energy restriction delivered as VLCD (25% estimated EI) on 2 consecutive days/week; no restriction on the other 5 days	2DW = −6.4 ± 11.2 kg ^e^	2DW = 20.8%
						CER 25% restriction below estimated requirements 7 days/week	CER = −5.6 ± 9.2 kg ^e^	CER = 13%
Varady et al., 2011 [47]	49 81.2% Female	35–65	25–39.9	12 weeks	Completer	AMDF 25% of baseline EI on fast days (meals provided), ad libitum at home on fed days	ADMF = −5.2 ± 4.0% ^e^	ADMF = 13.3%
						CER 75% of baseline EI daily (meals provided)	CER = −5.0 ± 4.9% ^e^	CER = 20%
						Exercise *Ad libitum* during the entire study; engaged in moderate intensity exercise program 3 times/week	Exercise = −5.1 ± 3.1% ^e^	Exercise = 20%
						Control *Ad libitum* during the entire study	Control = −0.2 ± 1.4% ^e^	Control = 20%
Harvie et al., 2013 [41]	115 100% Female	20–69^b^	24–45	12 weeks	Intent-to-Treat	2DW—Carbohydrate Restriction (CR) 30% EI of baseline energy requirements (high protein, moderate fat) with 40 g carbohydrate restriction on two consecutive days; eucaloric diet the other 5 days	2DW CR = 79.4 (95%CI 74.6–84.1) kg to 74.4 (95%CI 70.0–78.9) kg	2DW CR = 10.8%
						2DW—Carbohydrate Restriction + ad libitum Protein and Fat (CR+PF) 30% EI of baseline energy requirements and 40 g carbohydrate restriction with ad libitum protein and fat on two consecutive days; eucaloric diet the other 5 days	2DW CR + PF = 82.4 (95%CI 77.2–87.6) kg to 77.6 (95%CI 72.9–82.4) kg	2DW CR+PF = 18.4%
						CER 75% EI of baseline energy requirements daily	DER = 86 (95%CI 80.6–91.3) kg to 82.3 (95%CI 77.1–87.5) kg	DER = 17.5%
Carter et al., 2016 [42]	63 52.4% Female	≥18	≥27	12 weeks	Completer	2DW 1670–2500 kJ/day for 2 non-consecutive days each week, and 5 days of ad libitum intake	2DW = −6.2 ± 3.6%	2DW = 16.1%
						CER Continuous energy restriction diet of 5000–6500 kJ/day	CER = −5.6 ± 4.4%	CER = 21.8%
Catenacci et al., 2016 [25]	26 80% Female	18–55	≥30	8 weeks	Completer	ADF 100% energy restriction on fasting day, ad libitum on fed days. Meals provided	ADF = −8.8 ± 3.7% ^e^	ADF = 6.7%
						CER ~1675 kJ deficit per day. Meals provided	CER = −6.2 ± 3.1% ^e^	CER = 14.3%
Trepanowski et al., 2017 [48]	100 84% Female	18–64	25–40	26 weeks	Intent-to-Treat	ADMF 25% of baseline EI as lunch on fast days, 125% of baseline EI split between meals on fed days. Meals provided in first 3 months	ADMF = −6.8% (95%CI −9.1% to −4.5%)	ADF = 26.5%
						CER 75% of baseline EI split between three meals daily. Meals provided in first 3 months	CER = −6.8% (95% CI −9.1% to −4.6%)	CER = 17.1%
						Control No intervention; maintain baseline weight	As compared to control	Control = 19.4%
Carter et al., 2018 [43]	137 56.2% Female	≥18	≥27	52 weeks	Intent-to-Treat	2DW 2100–2500 kJ/day for 2 non-consecutive days each week, and 5 days of habitual eating	2DW = −6.8 ± 6.4 kg ^e^ (about −6.8%) ^c^	2DW = 28.6%
						CER Continuous energy restriction diet of 5000–6300 kJ/day	CER = −5.0 ± 7.1 kg ^e^ (about −4.9%) ^c^	CER = 31.3%
Conley et al., 2018 [45]	24 0% Female	55–75	≥30	26 weeks	Completer	2DW Restrict calorie intake to ~2500 kJ on two non-consecutive days per week; ad libitum intake on remaining five days	2DW = 5.3 ± 3.0 kg (5.5 ± 3.2%)	2DW = 8.3%
						CER Daily ~2100 kJ energy-restricted diet from average requirement	CER = 5.5 ± 4.3 kg (5.4 ± 4.2%)	CER = 0%
Schübel et al., 2018 [44]	150 50% Female	35–65	25–40	12 weeks	Intent-to-Treat	2DW Restriction at 80% weekly as 2 non-consecutive days with 75% energy restriction 5 days with no restriction	2DW = −7.1 ± 0.7% ^d^	2DW = 4.1%
						CER Continuous energy restriction at 80%	CER = −5.2 ± 0.6% ^d^	CER = 6.1%
						Control No energy restriction	Control = −3.3 ± 0.6% ^d^	Control = 1.9%
Sundfør et al., 2018 [46]	112 50% female	21–70	30–45	26 weeks	Intent-to-Treat	2DW ~1700/2500 kJ (female/male) energy restriction on two non-consecutive fasting days; ad libitum energy intake on remaining 5 days	2DW = −9.1 ± 5.0 kg (about −8.4%) ^c^	2DW = 1.9%
						CER Daily restriction of energy to match IER groups	CER = −9.4 ± 5.3 kg (about −8.7%) ^c^	CER = 3.4%
Hutchison et al., 2019 [49]	88 100% Female	35–70	25–42	10 weeks	Completer	ADMF 70 IF diet (32% EI on fast days and 100% EI on fed days) to equal 70% of calculated baseline energy requirements on three non-consecutive days per week	ADMF 70 = −5.4 ± 2.5 kg ^e^ (about −6.0%) ^c^	ADMF 70 = 12%
						ADMF 100 IF diet (37% of EI on fast days and 145% EI on fed days) to equal 100% of calculated baseline energy requirements on three non-consecutive days per week	ADMF 100 = −2.7 ± 2.5 kg ^e^ (about −3.2%) ^c^	ADMF 100 = 12%
						CER Continuous restriction at 70% of calculated baseline energy requirements daily	CER = −3.9 ± 2.0 kg ^e^ (about −4.4%) ^c^	CER = 7.7%
						Control 100% of calculated baseline requirements daily	Control = 0.4 ± 1.4 kg ^e^ (about 0.5%) ^c^	Control = 8.3%

Abbreviations: EI = energy intake; TDEE = total daily energy expenditure; ADF = alternate day fasting; ADMF = alternate day modified fasting; 2DW = fasting 2 days per week; CER = continuous energy restriction. ^a^ Reported attrition at end of intervention period, not at end of study periods, including follow-up observation or maintenance periods. ^b^ Age range of participants reflects baseline age of randomized participants. There was no age limit for recruitment in this study, but participants had to have gained ≥7 kg since the age of 20. ^c^ Estimated percent change in weight reported as absolute weight at end of the intervention compared to absolute weight at baseline. ^d^ Reported as individual log relative changes ± geometric SEM, with baseline values as the reference. ^e^ Reported as mean ±SD, converted from data in the manuscript by the authors for the purposes of this table.

**Table 2:** Changes in biomarkers of metabolic disease risk in randomized controlled weight loss trials of intermittent energy restriction (IER) compared to continuous energy restriction (CER).

Reference	Participants & Interventions	Measurement Conditions	Glycemic Outcomes	Lipid Outcomes	Other Biomarker Outcomes
Harvie et al., 2011 [40]	-Pre-menopausal women who were overweight or obese -2DW; CER	-Overnight fast, at least 5 days after the last partial energy restriction day in the 2DW group -In a subset of the 2DW group (n = 15), fasting blood samples were collected after 5 days of normal intake on the morning after a partial energy restriction day and after 2 days of normal intake	-Glucose (ND) -Greater decrease in insulin and increase in insulin sensitivity (HOMA-IR) in the 2DW group -Greater decreases in insulin sensitivity on the morning after a partial energy restriction day in a subset of the 2DW group	-Cholesterol (ND) -LDL (ND) -HDL (ND) -TGs (ND)	-CRP (ND) -Adiponectin (ND) -Leptin (ND) -Ketones (ND)
Varady et al., 2011 [47]	-Adults who were overweight or obese -ADMF; CER	12 h fasting blood samples	Not assessed	-Cholesterol (ND) -LDL (ND) -HDL (ND) -Greater decrease in TGs in the ADMF group	Not assessed
Harvie et al., 2013 [41]	-Pre-menopausal women -2DW + Carbohydrate Restriction; 2DW + Carbohydrate restriction and ad libitum protein and fat restriction; CER	Overnight fast, at least 5 days after the weekly 2 day partial energy restriction day in the 2DW groups	-Greater decrease in insulin and increase in insulin sensitivity (HOMA-IR) in the 2DW groups -HbA1c (ND) -Glucose (ND)	-Cholesterol (ND) -LDL (ND) -HDL (ND) -TGs (ND)	-IGF-1 (ND) -IL-6 (ND) -TNF-α (ND) -Ketones (ND) -Leptin (ND) -Adiponectin (ND)
Carter et al., 2016 [42]	-Adults with T2DM who were overweight or obese -2DW; CER	After an overnight fast (minimum of 8 h)	-HbA1c (ND)	Not assessed	Not assessed
Catenacci et al., 2016 [25]	-Adults who were overweight or obese -ADF; CER	After an overnight fast (minimum of 8 h)	-Glucose (ND) -Insulin (ND) -Insulin sensitivity (HOMA-IR; ND)	-Cholesterol (ND) -LDL (ND) -HDL (ND) -TGs (ND)	-Metabolic rate (ND) -Leptin (ND) -Ghrelin (ND) -BDNF (ND)
Trepanowski et al., 2017 [48]	-Adults who were overweight or obese -ADMF; CER	After a 12 h fast, the morning after a “feast” day for the ADMF group	-Glucose (ND) -Insulin (ND) -Insulin sensitivity (HOMA-IR; ND)	-Cholesterol (ND) -Greater increase in LDL in ADMF -HDL (ND) -TGs (ND)	-CRP (ND) -Homocysteine (ND)
Schübel et al., 2018 [44]	Adults who were overweight or obese -2DW; CER	Metabolic measures sampled day after fed day and an overnight fast	-Fasting glucose decreased more in 2DW than CER -Insulin (ND) -Insulin sensitivity (HOMA-IR; ND)	-Cholesterol (ND) -LDL (ND) -HDL (ND) -TGs (ND)	Not assessed
Sundfør et al., 2018 [46]	-Adults who were overweight or obese -2DW; CER	Blood samples were obtained following a minimum of a 10 h fast	Glucose (ND) HbA1c (ND)	-Cholesterol (ND) -LDL (ND) -HDL (ND) -TGs (ND) -Apo B (ND)	-CRP (ND) -Metabolic rate (ND)
Hutchison et al., 2019 [49]	-Female adults who were overweight or obese -ADMF 70; ADMF 100; CER	Metabolic measures performed after a fed and fasted day	-Greater decreases in glucose and insulin in ADMF 70 when measured after a fast day -Insulin sensitivity (euglycemic-hyperinsulinemic clamp) increased in ADMF 70 when measured after a fed day, but changes not different than CER -Insulin sensitivity (clamp) decreased in ADMF 70 when measured after a fasted day	-Greater decreases in fasting FFA, TC, LDL, and Tgs in ADMF 70 compared to CER, but ADMF 70 lost more weight -Differences in changes in cholesterol and LDL remained after adjusting for weight loss	Not assessed

Abbreviations: ADF = alternate day fasting; ADMF = alternate day modified fasting; 2DW = fasting 2 days per week; CER = continuous energy restriction; ND = no difference; HOMA-IR = Homeostatic Model Assessment of Insulin Resistance; LDL = low-density lipoprotein; HDL = high-density lipoprotein; TG = triglyceride; CRP = C-reactive protein; IGF-1 = insulin-like growth factor-1; IL-6 = interleukin-6; TNF-α = Tumor Necrosis Factor alpha; HbA1c = hemoglobin A1c; BDNF = brain-derived neurotrophic factor; Apo B = apolipoprotein B; FFA = free fatty acid.
